# Association between urinary 6β-hydroxycortisol/cortisol ratio and CYP3A5 genotypes in a normotensive population

**DOI:** 10.3892/etm.2012.842

**Published:** 2012-11-29

**Authors:** NAUSHAD RAIS, ARIF HUSSAIN, YOGESH KUMAR CHAWLA, KRISHAN K. KOHLI

**Affiliations:** 1 Department of Biotechnology, Manipal University, Dubai, United Arab Emirates;; 2 Departments of Hepatology, Postgraduate Institute of Medical Education and Research, Chandigarh, India; 3 Biochemistry, Postgraduate Institute of Medical Education and Research, Chandigarh, India

**Keywords:** pharmacogenetics, genetic polymorphism, cytochrome P4503A, North Indians, hypertension

## Abstract

Genetic polymorphism of genes involved in renal salt handling and arterial vessel tone is considered to be one of the causes of hypertension. Numerous reports suggest that cytochrome P4503A5 (CYP3A5) catalyzes 6β-hydroxylation of endogenous cortisol (CS), which is associated with sodium and water retention in the kidney and involved in the regulation of blood pressure. The purpose of the present study was to study the associations of single nucleotide polymorphisms in the CYP3A5 gene with the urinary 6β-hydroxycortisol/cortisol (6β-OH-CS/CS) ratio considered as quantitative phenotypes. CS measurements of three hundred (n=300) healthy, normotensive North Indian individuals was performed on morning spot urine samples by high-performance liquid chromatography. Furthermore, genotyping for CYP3A5*3 and CYP3A5*6 was performed by PCR-RFLP. The results indicated a unimodal distribution of CYP3A phenotypes in the North Indian population. In further analysis, all the phenotypes were distributed into three groups, demonstrating low (n=75), intermediate (n=150) and high CYP3A activity (n=75) based on CS and 6β-OH-CS levels and log 6β-OH-CS/CS ratios. The subjects in the low and high activity groups were genotyped for the CYP3A5*3 and *6 alleles. The present study demonstrated that the allele frequencies of CYP3A5*1 and *3 were 0.29 (95% CI, 0.22–0.36) and 0.71 (95% CI, 0.64–0.78), respectively. Notably, the frequency of normal homozygotes (CYP3A5*1/*1) was significantly higher in the high activity than the low activity group (11% vs. 5%). Similarly, the frequency of mutant homozygotes (CYP3A5*3/*3) was significantly higher in the low activity group than the high activity group (57% vs. 44%). The allele frequency of CYP3A5*3 was significantly higher in the low activity group (0.76) than the high activity group (0.67). The mean 6β-OH-CS/CS ratios were 110, 76 and 69 in wild-type homozygotes (n=12), heterozygotes (n=62) and mutant homozygotes (n=76), respectively. The difference between the normal and mutant homozygotes was statistically significant (P<0.05). The CYP3A5*6 allele was absent from all the subjects genotyped. This is the first study to report the genetic polymorphism of CYP3A5 in a North Indian population and its association with urinary 6β-OH-CS/CS ratio reflecting the CYP3A phenotypes.

## Introduction

The cytochrome P4503A (CYP3A) subfamily of enzymes are steroid 6β-hydroxylases which convert cortisol (CS) to 6β-hydroxycortisol (6β-OH-CS) and corticosterone to 6β-hydroxycorticosterone (1–3). Early studies on this subfamily of CYPs focused on the CYP3A4 isoform since it appeared to be predominantly expressed in human liver. However, in 2001 Kuehl *et al*(4) reported the expression of CYP3A5 in the livers of 50% of African-Americans but only one-third of Caucasians. It is now clear that CYP3A5 may also contribute significantly, although variably, to drug metabolism (5). CYP3A5 expression is predominant in the kidney, limited to the proximal tubule and affected by the CYP3A5*1/*3 polymorphism (6). The kidney is capable of CS 6β-hydroxylation, but only in individuals who express CYP3A5 (7). Animal (2–3) and *in vitro*(8–9) studies have reported a correlation of the expression of CYP3A enzymes with sodium reabsorption and blood pressure (BP). Thus, genetic polymorphism in CYP3A5 may affect endogenous CS metabolism in the proximal renal tubule (10) that may ultimately affect BP, likely through sodium and water retention. However, reports concerning the association of CYP3A5 genetic polymorphism with BP or hypertension have been largely inconsistent in humans (11–15). We have previously reported the absence of CYP3A4 genetic polymorphism in North Indian individuals and its correlation with the urinary 6β-hydroxy cortisol/cortisol (6β-OH-CS/CS) ratio (16).

In the present study, healthy normotensive subjects were phenotyped for CYP3A activity by assaying the urinary 6β-OH-CS/CS ratio and genotyped for CYP3A5*3 and CYP3A5*6 to establish whether a correlation exists in the North Indian population.

## Materials and methods

### Reagents

Bangalore Genei Pvt. Ltd. (Bangalore, India) supplied Taq DNA polymerase, PCR buffer, dNTPs and *Hin*fI. New England Biolabs, Inc. (Beverly, MA, USA) supplied *Xcm*I, *Bfa*I, *Dde*I and *Hpy*CH4III. MBI Fermentas (Hanover, MD, USA) supplied *Cla*I, *Mbo*II and *Bsm*A1. Operon Technologies, Inc. (Alameda, CA, USA) synthesized the primers. Sigma Chemical Co. (St. Louis, MO, USA) supplied CS and 6β-OH-CS. Ranbaxy Fine Chemicals Ltd. (New Delhi, India) supplied the high-performance liquid chromatography (HPLC) solvents.

### Subjects and sample collection

Three hundred (n=300) healthy volunteers aged 20–50 years who were normotensive (BP≤120), non-smokers, non-alcoholics and not on any medication for the previous two weeks were selected for the study. Written consent along the Helsinki on experimentation involving humans was obtained from each volunteer. The present study was performed at the Department of Biochemistry and approved by the Ethics Committee of the Postgraduate Institute of Medical Education and Research (Chandigarh, India).

Morning spot urine samples were collected between 8 and 9 am in 20-ml screw-tight glass vials. These glass vials were washed with nitric acid and baked in an oven at 150°C for 3 h. Urine samples were brought to the laboratory as soon as possible and stored at −20°C. Blood samples (5 ml) from the subjects selected for genotyping were collected in a vial containing 875 μl acid citrate dextrose.

### Phenotyping

A total of 300 North Indian individuals were phenotyped for CYP3A by measuring CS and 6β-OH-CS levels in urine by HPLC as described previously (16).

### Genotyping

Blood (5 ml) was collected in a vial containing ACD (0.48% citric acid, 1.32% sodium citrate and 1.47% dextrose) from 150 subjects (75 demonstrating low and 75 demonstrating high CYP3A activity). DNA was isolated (17) and stored in a refrigerator until use. The PCR conditions, primers and restriction endonucleases to diagnose CYP3A5*3 and CYP3A5*6 were as described previously (18) and are presented in Table I. The amplified 200-bp DNA fragment for CYP3A5*3 contains one *Dde*I site. This mutation results in the creation of an additional *Dde*I site. *Dde*I digestion of DNA from a normal homozygote (CYP3A5*1/*1) produces 133- and 67-bp fragments, while a heterozygote (CYP3A5*1/*3) has 133-, 108-, 67- and 25-bp fragments and a mutant homozygote (CYP3A5*3/*3) has 108-, 67- and 25-bp fragments. The amplified 236-bp DNA fragment for CYP3A5*6 contains two *Dde*I sites. This mutation results in the loss of one *Dde*I site. The *Dde*I digestion of DNA from a normal homozygote (CYP3A5*1/*1) produces 103-, 77-, 31- and 25-bp fragments, while a heterozygote (CYP3A5*1/*6) has 128-, 103-, 77-, 31- and 25-bp fragments and a mutant homozygote (CYP3A5*6/*6) has 128-, 77- and 31-bp fragments.

### Statistical analysis

Analysis of the interindividual variations in the metabolism of CS was expressed by computing a histogram with log 6β-OH-CS/CS ratio on the x-axis and the number of subjects on the y-axis. The CYP3A5 genotypes and allele frequencies were compared by the Chi-square test. Data were analyzed by nonparametric one-way Kruskal-Wallis ANOVA followed by Mann-Whitney U tests. P<0.05 was considered to indicate a statistically significant difference.

## Results

### Phenotype analysis

**Figure 1.**
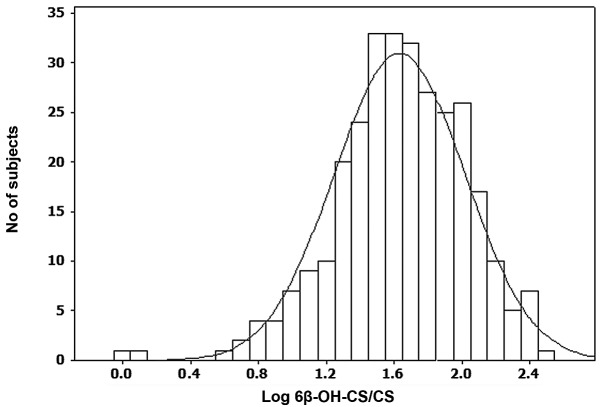
Frequency distribution histogram of log 6β-OH-CS/CS in 300 North Indian individuals. Log 6β-OH-CS/CS and number of subjects are shown on the x- and y-axis, respectively. Phenotyping was performed by measuring CS and 6β-OH-CS in morning spot urine samples by HPLC. HPLC was performed on a Waters HPLC system, using a mobile phase consisting of 70% 50 mM KH_2_PO_4_, 10 mM acetic acid and 30% acetonitrile (pH 4.0). The flow rate was maintained at 1 ml/min. The eluant was monitored at 244 nm. HPLC, high-performance liquid chromotography; 6β-OH-CS/CS, 6β-hydroxycortisol/cortisol ratio.

CYP3A phenotype data was plotted on the x-axis and number of subjects on the y-axis to generate frequency distribution histogram (Fig. 1) which demonstrated a unimodal distribution with respect to CYP3A activity. The mean 6β-OH CS/CS ratio was 61 (95% CI, 55–67). On the basis of CYP3A activity, the subjects were divided into three groups demonstrating low (n=75), intermediate (n=150) and high (n=75) CYP3A activity (Table II). The mean CS concentrations were 197, 124 and 58 ng/ml in the urine of the low, intermediate and high CYP3A activity groups, respectively, and the mean 6β-OH-CS concentrations were 2,931, 5,596 and 7,446 ng/ml in the urine of the low, intermediate and high CYP3A activity groups, respectively. The 6β-OH-CS/CS ratio in urine was 16 in the low, 47 in the intermediate and 135 in the high CYP3A activity groups (Table II). CS levels were statistically significant lower in the intermediate and high CYP3A activity groups than in the low CYP3A activity group, whereas 6β-OH-CS levels, the 6β-OH-CS/CS ratio and log 6β-OH-CS/CS were statistically significantly higher (P<0.01). The 6β-OH-CS/CS and log 6β-OH-CS/CS ratios in the high CYP3A activity group were 8-fold and 1.84-fold higher, respectively, than those in the low CYP3A activity group. CS levels were also statistically significantly lower (P<0.01) in the high CYP3A activity group than in the intermediate CYP3A activity group, whereas 6β-OH-CS/CS and log 6β-OH-CS/CS ratios were statistically significantly higher (P<0.01; Table II).

### Genotype analysis

**Figure 2.**
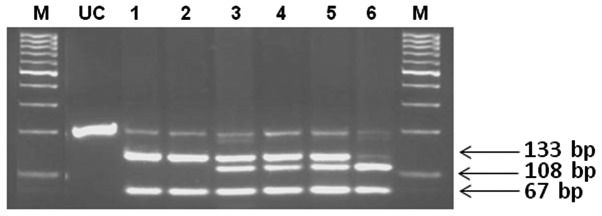
PCR based diagnostic test of CYP3A5*3. Genomic DNA was isolated, amplified by PCR and digested with *Dde*I, followed by electrophoresis on a 3% agarose gel containing ethidium bromide. Lane M represents a 100-bp DNA ladder. Lane UC represents the uncut 200-bp PCR product amplified for CYP3A5*3. Lanes 1 and 2 show 133- and 67-bp fragments representing samples from normal homozygotes (CYP3A5*1/*1). Lanes 3 to 5 show 133-, 108- and 67-bp fragments representing samples from heterozygotes (CYP3A5*1/*3). Lane 6 shows 108- and 67-bp fragments representing samples from mutant homozygotes (CYP3A5*3/*3). CYP3A5, cytochrome P4503A5.

**Figure 3.**
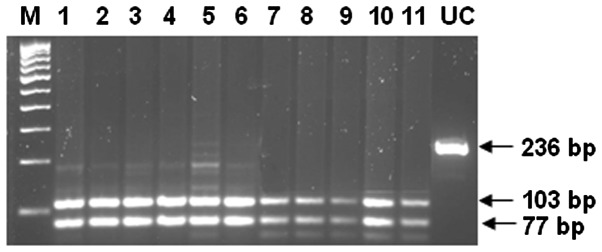
PCR based diagnostic test of CYP3A5*6. Genomic DNA was isolated, amplified by PCR and digested with *Dde*I, followed by electrophoresis on a 3% agarose gel containing ethidium bromide. Lane M represents a 100-bp DNA ladder. Lane UC represents the uncut 236-bp PCR product. Lanes 1 to 11 show 103- and 77-bp fragments representing samples from normal homozygotes (CYP3A5*1/*1). CYP3A5, cytochrome P4503A5.

**Figure 4.**
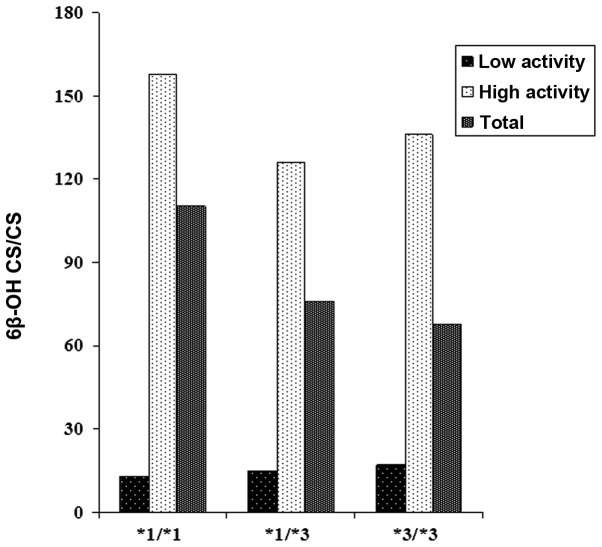
Bar diagram showing mean 6β-OH-CS/CS ratios in North Indian individuals with various CYP3A5*3 genotypes. The activity of CYP3A was assayed by measuring CS and 6β-OH-CS in morning spot urine samples by HPLC. HPLC was performed using a mobile phase consisting of 70% 50 mM KH_2_PO_4_, 10 mM acetic acid and 30% acetonitrile (pH 4.0). The flow rate was maintained at 1 ml/min. The eluant was monitored at 244 nm. Subjects exhibiting low and high CYP3A activity were genotyped for CYP3A*3 by PCR-RFLP as described in Materials and methods. CYP3A5, cytochrome P4503A5; HPLC, high-performance liquid chromotography; 6β-OH-CS/CS, 6β-hydroxycortisol/cortisol ratio.

The CYP3A5 genotypes were determined by PCR-RFLP (Figs. 2 and 3). The correlations between the CYP3A phenotypes and CYP3A5 genotypes in the low and high CYP3A activity groups are shown in Table III and Fig. 4. Normal homozygotes (CYP3A5*1/*1), heterozygotes (CYP3A5*1/*3) and mutant homozygotes (CYP3A5*3/*3) in the high CYP3A activity group exhibited statistically significantly lower CS levels when compared with the low CYP3A activity group, whereas statistically significantly higher 6β-OH-CS, 6β-OH-CS/CS and log 6β-OH-CS/CS ratios were observed. When the 6β-OH-CS/CS ratios of the genotypes were compared within the high CYP3A activity group, heterozygotes (CYP3A5*1/*3) and mutant homozygotes (CYP3A5*3/*3) demonstrated 20 and 14% decreases, respectively, compared with the normal homozygotes (CYP3A5*1/*1). These decreases were much higher (30 and 37%, respectively) when the 6β-OH-CS/CS ratios in heterozygotes (CYP3A5*1/*3) and mutant homozygotes (CYP3A5*3/*3) were compared with normal homozygotes (CYP3A5*1/*1) in the total study population. Although the results are not statistically significant, these suggest that CYP3A5*3 reduced the urinary 6β-OH-CS/CS ratios and, as such, CYP3A5*3 is a debilitating allele.

The distribution of the genotypes in the low and high CYP3A activity groups is shown in Table IV. Out of 150 subjects genotyped for CYP3A5*3, 12 (8%) were normal homozygotes (CYP3A5*1/*1), 62 (41%) were heterozygotes (CYP3A5*1/*3) and 76 (51%) were mutant homozygotes (CYP3A5*3/*3). Thus, the frequencies of CYP3A5*1 and CYP3A5*3 were 0.29 (95% CI, 0.22–0.36) and 0.71 (95% CI, 0.64–0.78) in 150 North Indian individuals (Table IV). Out of 75 subjects genotyped for CYP3A5*3 in the low CYP3A activity group, 4 (5%) were normal homozygotes (CYP3A5*1/*1), 28 (37%) were heterozygotes (CYP3A5*1/*3) and 43 (57%) were mutant homozygotes (CYP3A5*3/*3; Table IV). Thus, the frequencies of CYP3A5*1 and CYP3A5*3 were 0.24 and 0.76, respectively in the low CYP3A activity group. Out of 75 subjects genotyped for CYP3A5* 3 in the high CYP3A activity group, 8 (11%) were normal homozygotes (CYP3A5*1/*1), 34 (45%) were heterozygotes (CYP3A5*1/*3) and 33 (44%) were mutant homozygotes (CYP3A5*3/*3; Table IV). Thus, the frequencies of CYP3A5*1 and CYP3A5*3 were 0.33 and 0.67, respectively, in the high CYP3A activity group. There were 30% more mutant homo-zygotes (CYP3A5*3/*3) and 14% more CYP3A5*3 alleles in the low CYP3A activity group than in the high CYP3A activity group. These observations support the previous suggestion that CYP3A5*3 reduced the activity of CYP3A, as its occurrence was higher in the low CYP3A activity group and lower in the high CYP3A activity group (Fig. 4).

## Discussion

The single nucleotide polymorphisms (SNPs) reported in CYP3A4 in Caucasians are not detrimental and are present at low frequencies to account for variation in CYP3A activity. CYP3A5*3 and CYP3A*6 have been shown to drastically reduce CYP3A activity. Hence, in the present study the correlation between CYP3A activity and CYP3A5*3 and CYP3A*6 was studied in a North Indian population. Since the population demonstrated a unimodal distribution with respect to CYP3A activity (Fig. 1), the individuals were divided into three groups of low, intermediate and high CYP3A activity (Table II). The CYP3A activity in heterozygotes (CYP3A5*1/*3) and mutant homozygotes (CYP3A5*3/*3) was not different from that in normal homozygotes (CYP3A5*1/*1) in the low CYP3A activity group, but exhibited 20 and 14% decreases, respectively, from that in normal homozygotes in the high CYP3A activity group. These decreases increased to 31 and 37% in the total study population. Although the data were not statistically significant, it suggested that CYP3A5*3 reduced the CYP3A activity (Table III). This was further supported by the observation that mutant homozygotes (CYP3A5*3/*3) were present at high frequency in the low CYP3A activity group and low frequency in the high CYP3A activity group (Table IV). The results are statistically insignificant due to the fact that CYP3A4 and CYP3A5 contribute towards CYP3A activity and, while CYP3A4 is expressed in all the livers, hepatic CYP3A5 is polymorphically expressed in ∼30% of Causcasian, Asian and Hispanic individuals and >50% African Americans (4). The corresponding information is not available for an Indian population, but it may be estimated from the data generated in the present study that hepatic CYP3A5 is expressed in ∼50% North Indian individuals (normal homozygotes plus heterozygotes; Table IV). However, this must be substantiated by assaying the CYP3A5 protein content by immunochemical techniques and is constrained by the availability of human livers.

CYP3A5 is important due to the differential metabolism of specific substrates, despite a substantial overlap with the substrate specificity of CYP3A4. CYP3A5 metabolizes cyclosporine slower than CYP3A4 and produces only one metabolite, MI, whereas CYP3A4 produces two additional metabolites, AM9 and AMN4 (19). Mugundu *et al*(20) assessed the contributions of CYP3A4 and CYP3A5 and examined the impact of the CYP3A5 genotype on the formation of α-hydroxytamoxifen (α-OHT) and N-desmethyltamoxifen (N-DMT) from tamoxifen and suggested that CYP3A5 expression may affect the formation of N-DMT but not that of α-OHT. Differences have also been reported in the rate of the metabolism of testosterone, progesterone and androstenedione by CYP3A4 and CYP3A5 (19). Most significantly, an association exists between tacrolimus, an immunosuppressant with a narrow therapeutic index, and the CYP3A5 genotypes. Eight studies performed on tacrolimus with regard to CYP3A5 genotypes in organ transplant patients in various ethnic groups (21) demonstrated a direct correlation between the tacrolimus dose required to reach a predetermined trough concentration and CYP3A5 genotypes. Mutant homozygotes (CYP3A5*3/*3) required less tacrolimus to reach the trough levels than normal homozygotes (CYP3A5*1/*1), as the former have lower metabolic activity than the latter. In support of this, baculovirus-expressed CYP3A5 metabolized tacrolimus to 13-O-demethyltacrolimus, the main metabolite, at a higher rate compared with CYP3A4 and CYP3A7 (22). Human hepatic microsomes from low and high CYP3A expressors metabolized tacrolimus at different rates. The high expressors metabolized tacrolimus at a faster rate than the low expressors. It must be noted that the 15 human hepatic microsomes used in this study had been phenotyped by CS-6β-hydroxylase (22), thus demonstrating that the metabolism of tacrolimus and CS are closely correlated.

Another important hypothesis concerning the CYP35 genotype is that it plays role in BP since CYP3A5 and not CYP3A4 is expressed in extrahepatic tissues, particularly the kidney (23). As mentioned previously, CYP3A5 metabolizes CS to 6β-OH-CS. The local metabolism of CS to 6β-OH-CS increases the metabolite concentration in the kidney. The metabolites 6β-OH-CS and 6β-OH corticosterone act as mineralocorticoids, which lead to hypertension due to electrolyte and water retention in kidney. Levels of 6β-OH-CS have been shown to be elevated in hypertensive individuals (24). Moreover, African American normal homo-zygotes (CYP3A5*1/*1) exhibited higher systolic BP, mean arterial pressure and creatinine clearance compared with heterozygotes (CYP3A5*1/*3) (23). These observations were confirmed in a larger cohort (25). It is notable that all three; normal homozygotes (CYP3A5*1/*1) (4), hypertension (26) and salt sensitivity (27) are simultaneously higher in African Americans. This information is lacking for an Indian population and should be investigated.

The normal homyzygote frequency (CYP3A5*1/*1) was 8%, heterozygote frequency (CYP3A5*1/*1) was 41% and mutant homozygote frequency (CYP3A5*3/*3) was 51%. This data agrees with the pooled data from other studies which demonstrated the frequency of normal homozygotes (CYP3A5*1/*1) to be 7.3%, heterozygotes (CYP3A5*1/*1) to be 39.7% and mutant homozygotes (CYP3A5*3/*3) to be 53.0% in an Asian population (28). According to these studies, CYP3A5 expressors should be ∼50% of the population, whereas CYP3A5 expressors have been reported to be 69% (normal homozygotes plus heterozygotes) in an Indian population (18). Accordingly, the CYP3A5*3 allele frequency reported in Indian individuals was 0.59 (18), lower than the 0.71 reported in the present study. This is due to the fact that the present cohort included North Indians, whereas migrants living in Singapore, who were an admixture of various ethnic groups from India, were selected in the earlier study (18). Heterogeneity within the Indian population has been documented. We previously reported the absence of CYP2C19*3 in North Indian individuals (29), whereas its frequency in South Indian individuals has been reported to be 0.022 (30). It would be prudent to give due consideration to the various ethnic groups within India while studying genetic polymorphism of CYPs.

## Figures and Tables

**Table I.** Primers, PCR conditions, REs and diagnostic DNA fragments for genotyping CYP3A5 alleles.

Allele	Primers	PCR (35 Cycles)	RE	DNA Fragments
3A5*3	FP: 5′-CTTAAAGAGCTCTTTTGTCTCTCA-3′	45 sec, 94°C	*Dde*I	AF 200 NH 133,67 HE 133, 108, 67, 25 MH 108, 67,25
	RP: 5′-CCAGGAAGCCAGACTTTGAT-3′	45 sec, 69°C		
		30 sec, 72°C		
3A5*6	FP: 5′-GTGGGTTTCTTGCTGCATGT-3′	45 sec, 94°C	*Dde*I	AF 236 NH 103, 7,31,25 HE 128, 103,77,31,25 MH 128 77, 31
	RP: 5′-GCCCACATACTTATTGAGAG-3′	45 sec, 69°C		
		30 sec, 72°C		

RE, restriction endonuclease; CYP3A5, cytochrome P4503A5; FP, forward primer; RP, reverse primer; AF, amplified fragment; NH, normal homozygote; HE, heterozygote; MH, mutant homozygote.

**Table II.** CYP3A phenotype parameters in the low, intermediate and high CYP3A activity groups.

Urine parameter	Low CYP3A activity group (n=75)	Intermediate CYP3A activity group (n=150)	High CYP3A activity group (n=75)
CS (ng/ml)	197±118	124±92^a^	58±56^ab^
6β-OH-CS (ng/ml)	2931±2211	5596±4210^a^	7446±7845^ac^
6β-OH-CS/CS	16±6	47±15^a^	135±53^ab^
Log 6β-OH-CS/CS	1.14±0.26	1.65±0.14^a^	2.10±0.15^ab^

Data are the mean ± SD and were analyzed by nonparametric one-way Kruskal-Wallis ANOVA followed by Mann-Whitney U tests.

a All parameters in the intermediate and high CYP3A activity groups exhibited statistically significant differences (P<0.01) when compared with the corresponding paramaters for the low CYP3A activity group.

b The CS level in the high CYP3A activity group was statistically significantly lower than that in the intermediate CYP3A activity group, whereas 6β-OH-CS/CS and log 6β-OH-CS/CS in the high activity group were statistically significantly higher.

c The 6β-OH-CS level in the high activity group was not significantly different from that in the intermediate CYP3A activity group. CYP3A5, cytochrome P4503A5; CS, cortisol; 6β-OH-CS, 6β-hydroxycortisol.

**Table III.** Correlation between CYP3A phenotypes and CYP3A5 genotypes.

CYP3A5 genotypes	Low CYP3A activity group	High CYP3A activity group	P-value	Total
CS (ng/ml urine)				
CYP3A5*1/*1	182±107	63±64	0.048	103±96
CYP3A5*1/*3	225±113	55±45	0.0001	132±118
CYP3A5*3/*3	180±121	57±65	0.0003	126±117
6β-OH-CS (ng/ml urine)				
CYP3A5*1/*1	2581±1811	9231±8738	0.283	7015±7759
CYP3A5*1/*3	3210±2364	6681±6675	0.002	5114±5442
CYP3A5*3/*3	2781±2169	7290±8739	0.0001	4739±6347
6β-OH-CS/CS (urine)				
CYP3A5*1/*1	13.5±3.2	158±65	0.004	110±80
CYP3A5*1/*3	14.7±6.8	126±57	0.0001	76±70
CYP3A5*3/*3	16.5±6.4	136±46	0.0001	69±67
Log 6β-OH-CS/CS (urine)				
CYP3A5*1/*1	1.11±0.11	2.17±0.18	0.004	1.82±0.54
CYP3A5*1/*3	1.10±0.30	2.07±0.66	0.0001	1.63±0.54
CYP3A5*3/*3	1.17±0.25	2.11±0.13	0.0001	1.58±0.51

Data represent the mean ± SD and were analyzed by nonparametric one-way Kruskal-Wallis ANOVA followed by Mann-Whitney U tests. P-values are for CYP3A phenotype parameters of CYP3A5 genotypes in the high CYP3A activity group compared with the respective CYP3A phenotype parameters in the low CYP3A activity group. All parameters with the exception of CS and 6β-OH-CS in normal homozygotes (CYP3A5*1/*1) in the high CYP3A activity group were statistically significant when compared with the low CYP3A activity group. CS, 6β-OH-CS, 6β-OH-CS/CS and log 6β-OH-CS/CS did not exhibit statistically significant differences when heterozygotes (CYP3A5*1/*3) and mutant homozygotes (CYP3A5*3/*3) were compared with normal homozygotes (CYP3A5*1/*1) or among themselves. CYP3A5, cytochrome P4503A5; CS, cortisol; 6β-OH-CS, 6β-hydroxycortisol.

**Table IV.** CYP3A5 genotype and allele frequency in low and high CYP3A activity groups of North Indian individuals.

CYP3A5 genotypes/alleles	Low CYP3A activity group (n=75)	High CYP3A activity group (n=75)	Total (n=150)
Genotypes			
CYP3A5*1/*1	4 (5 %)	8 (11 %)	12 (8 %)
CYP3A5*1/*3	28 (37 %)	34 (45 %)	62 (41 %)
CYP3A5*3/*3	43 (57 %)	33 (44 %)	76 (51 %)
Alleles			
CYP3A5*1	0.24	0.33	0.29
CYP3A5*3	0.76	0.67	0.71

Data was analyzed by the Chi-square test. CYP3A5 genotypes and allele frequencies in the high CYP3A activity group were not statistically different from those in the low CYP3A activity group.
